# Augmenting E Protein Activity Impairs cDC2 Differentiation at the Pre-cDC Stage

**DOI:** 10.3389/fimmu.2020.577718

**Published:** 2020-12-18

**Authors:** Sandra Bajana, Kevin Thomas, Constantin Georgescu, Ying Zhao, Jonathan D. Wren, Susan Kovats, Xiao-Hong Sun

**Affiliations:** ^1^ Program in Arthritis and Clinical Immunology, Oklahoma Medical Research Foundation, Oklahoma City, OK, United States; ^2^ Program in Genes and Human Diseases, Oklahoma Medical Research Foundation, Oklahoma City, OK, United States

**Keywords:** E protein, cDC1, cDC2, pre-cDC, IRF4, IRF8

## Abstract

Dendritic cell (DC) specification and differentiation are controlled by a circuit of transcription factors, which regulate the expression of DC effector genes as well as the transcription factors themselves. E proteins are a widely expressed basic helix-loop-helix family of transcription factors whose activity is suppressed by their inhibitors, ID proteins. Loss-of-function studies have demonstrated the essential role of both E and ID proteins in different aspects of DC development. In this study, we employed a gain-of-function approach to illustrate the importance of the temporal control of E protein function in maintaining balanced differentiation of conventional DC (cDC) subsets, cDC1 and cDC2. We expressed an E protein mutant, ET2, which dimerizes with endogenous E proteins to overcome inhibition by ID proteins and activate the transcription of E protein targets. Induction of ET2 expression at the hematopoietic progenitor stage led to a dramatic reduction in cDC2 precursors (pre-cDC2s) with little impact on pre-cDC1s. Consequently, we observed decreased numbers of cDC2s in the spleen and lung, as well as in FLT3L-driven bone marrow-derived DC cultures. Furthermore, in mice bearing ET2, we detected increased expression of the IRF8 transcription factor in cDC2s, in which IRF8 is normally down-regulated and IRF4 up-regulated. This aberrant expression of IRF8 induced by ET2 may contribute to the impairment of cDC2 differentiation. In addition, analyses of the transcriptomes of splenic cDC1s and cDC2s revealed that ET2 expression led to a shift, at least in part, of the transcriptional profile characteristic of cDC2s to that of cDC1. Together, these results suggest that a precise control of E protein activity is crucial for balanced DC differentiation.

## Introduction

Dendritic cells (DCs) orchestrate a variety of immune responses and are thus important players in responses to microbial infection, tumor immunity and autoimmunity (1). Subsets of DCs are categorized as class 1 and class 2 conventional dendritic cells (cDC1 and cDC2), as well as plasmacytoid dendritic cells (pDC), each with specialized functions (2, 3). While cDC1s generally promote type 1 immune responses against intracellular pathogens, cDC2s promote types 2 and 3 reactions triggered by parasites, extracellular bacteria and fungi. pDCs are stimulated by intracellular nucleic acids arising during viral infection and produce large amounts of type I interferon. Despite their diverse functions, all DCs originate in the bone marrow mostly through common dendritic cell progenitors (CDP) (4–6), although some lymphoid progenitors are also known to give rise to pDCs (7). CDPs then branch into precursors of cDC (pre-cDC) and pDC (pre-pDC) in the bone marrow. Recently, pre-cDCs have been further divided into pre-cDC1 and pre-cDC2, which circulate in the blood and differentiate into their respective cDC classes in the periphery (8, 9).

The transcriptional regulation of DC ontogeny has been extensively studied. Two transcription factors, IRF8 and IRF4, are instrumental for the differentiation and function of cDC1 and cDC2 subsets, respectively. Other transcription factors including BATF3, NFIL3, BCL6 and ID2 have been shown to be essential for cDC1 production (3). However, these factors mostly act through regulation of *Irf8* transcription. Besides being a terminal selector of cDC1, IRF8 plays critical roles in the maintenance of DC progenitors (9, 10). In contrast, as cDC2s differentiate from pre-cDC2s, they down-regulate *Irf8* and up-regulate *Irf4* (11). Whether shutting off IRF8 is a pre-requisite of cDC2 maturation is not entirely clear, but it has been shown that over expression of IRF8 impairs cDC2 differentiation (9). How IRF8 inhibits cDC2 differentiation is not known. Given their structural similarities, it is possible that IRF8 antagonizes the function of IRF4 or competes for a common binding partner such as PU.1. Alternatively, a balance between the amounts of IRF4 and IRF8 influences cDC2 differentiation.

Members of the helix-loop-helix family of transcription factors also influence DC differentiation at multiple checkpoints. These regulators include E protein transcription activators encoded by the E2A, HEB and E2-2 genes and their dominant-negative inhibitors called ID proteins (ID1-4) (12, 13). Although all E proteins are expressed at tonic levels, E2-2 is dramatically up-regulated and instrumental for pDC formation (14). In contrast, ID2 expression is increased and essential for cDC1 production (15, 16). Prior to DC specification, E proteins bind to the regulatory sequences of *Irf8* and activate its transcription (17). E protein activities are then repressed by the expression of ID2, which is controlled by another transcription factor, ZEB2 (15). This coordinated regulation of E protein activity may be necessary for balanced cDC differentiation. In the presence of high levels of ID2 in cDC1s, the maintenance of high levels of IRF8 relies on the cDC1-specific expression of BATF3 whereas *Irf8* remains silenced in cDC2s (9). Therefore, sustained E protein activity could disturb the balance of DC precursors in the bone marrow, leading to skewed proportions of DC subsets in the periphery.

To test this hypothesis, we specifically expressed our gain of E protein function mutant in hematopoietic progenitors and committed DCs and examined the impact on the distribution of cDC1s and cDC2s in lymphoid and peripheral tissue. Sustained E protein activity led to a reduction in cDC2 numbers in both the spleen and lung. Moreover, analyses of pre-cDC progenitors in the bone marrow revealed that a subset of pre-cDC2s that express CD11b was selectively diminished in mice with elevated E protein function. Gain of E protein function also led to impaired cDC2 differentiation in bone marrow cultures supported by FLT3 ligand. These phenotypes were accompanied by high levels of IRF8 expression in pre-cDC2s and cDC2s that normally express low IRF8 levels. These studies illustrate the crucial role of E proteins at multiple checkpoints of DC differentiation and the importance of the dynamic regulation of E proteins for maintaining the balance of DC diversity.

## Materials and Methods

### Mouse Models

Rosa26-stop-ET2 was generated by knocking ET2 along with IRES-EGFP into the Rosa26 locus downstream of its promoter as previously described (18). CD11c-Cre and Vav1-iCre expressing mice were purchased from Jackson Laboratory (Bar Harbor, ME). ET2^CD11c^ mice are homozygous for the ET2 allele whereas ET2^Vav^ mice have one ET2 allele. Littermate Cre^–^ ET2 mice served as controls.

### Cell Isolation

A single-cell suspension from the spleen was obtained after incubation for 30 min at 37°C in HBSS buffer with calcium and magnesium with Collagenase D (1mg/ml) and DNase (0.1 mg/ml). Lungs were perfused with PBS through the right ventricle, cut into small pieces and enzymatically digested by 45 min incubation at 37°C with Collagenase D (1mg/ml) and DNAse (0.1mg/ml) in HEPES buffer containing 10 mM HEPES-NaOH pH 7.4, 150 mM NaCl, 5 mM KCl, 1 mM MgCl_2_, 1.8 mM CaCl_2_. Cell suspension was passed through a 70 μm cell strainer, followed by RBC lysis in buffered ammonium chloride.

### Flow Cytometry and Cell Sorting

All antibodies were purchased from BioLegend unless specified otherwise: anti- I-A/I-E (M5/114.15.2), anti-CD11c (N418), anti-Siglec H (551), anti-CD8a (53-6.7), anti CD4 (GK1.5), anti-CD45R/B220 (RA3-6B2), anti-CD11b (M1/70), anti-CD103 (2E7), anti-CD24 (M1/69), anti-CD135 (A2F10), anti-CD172a/SIRPα (P84), anti-Ly-6C (HK1.4), anti-IRF4 (Thermofisher; 3E4) anti-IRF8 (Thermofisher; V3GYWCH), CD88/C5aR (20/70), and anti-CD26/DPP-4 (H194-112). Antibodies in the lineage (Lin) cocktail are anti-B220 (RA3-6B2), anti-CD3 (17A2), anti-CD19 (6D5), anti-NK-1.1 (PK136), anti-Ly6G (1A8) and anti-TER119 (TER119).

Cell sorting was performed on a FACSAria II (BD Biosciences), and flow cytometric analysis was performed on a LSR-II (BD Biosciences). Intracellular staining of transcription factors was done using Foxp3 Staining Buffer kit (eBioscience).

### *In Vitro* Bone Marrow Culture

Bone marrow-derived DC culture driven by FLT3 ligand was carried out essentially as described (19). Briefly, bone marrow cells were enriched for progenitors by depleting differentiated cells with purified antibodies against CD11b, B220, CD3, CD5, Ly6G and TER119 (Biolegend), and anti-rat IgG-conjugated magnetic beads (Qiagen). Cells in the supernatant were washed and resuspended in RPMI1640 medium supplemented with 10% fetal calf serum, 50 ng/ml stem cell factor and 100 ng/ml FLT3 ligand. Cells were maintained in the same medium for 9 days by replacing half of the medium with fresh cytokine on day 2 and day7. Cells were then harvested and stained with antibodies against CD11c, MHCII, B220, SIRPα, SIGLEC-H and CD24.

### Analyses of RNA Sequencing Data

After the FASTQ files were generated from the RNA-sequencing run, the 5’ and 3’ ends of the raw reads were processed using Trimmomatic (20) to remove low-quality bases and adapter sequences. These processed RNA-seq reads were then aligned to the *Mus musculus* reference genome (GRCm38/mm10) using STAR v.2.4.0h (21). HTSeq v.0.5.3p9 (22) was used to determine gene-level read counts according to the annotations in GENCODE Release M10 (GRCm38). Read-count normalization and differentially expressed analyses was performed using the edgeR package from Bioconductor. Only autosomal genes coding for lncRNAs, miRNAs, and protein-coding mRNAs were selected for further analyses. The voom function within the software package limma was used to normalize expression values and evaluate which transcripts were differentially expressed (DE) between conditions. The statistical significance of DE transcripts was assessed using moderated t-statistics, and p-values were adjusted for multiple testing using false discovery rate (FDR). Unless otherwise specified, only DE transcripts with at least two fold change in expression and a FDR < 0.05 were selected. The final set of DE transcripts was assessed using Ingenuity Pathway Analysis (IPA, QIAGEN, Redwood City CA) to explore significant gene networks and pathways.

### Statistical Analysis

Statistical analysis was performed using Prism 6 (GraphPad Software). Specific tests applied are indicated in each figure legend. Data are presented as mean +/- SEM.

## Results

### Sustained E Protein Activity Leads to a Reduction in cDC2s in the Spleen

**Figure 1:**
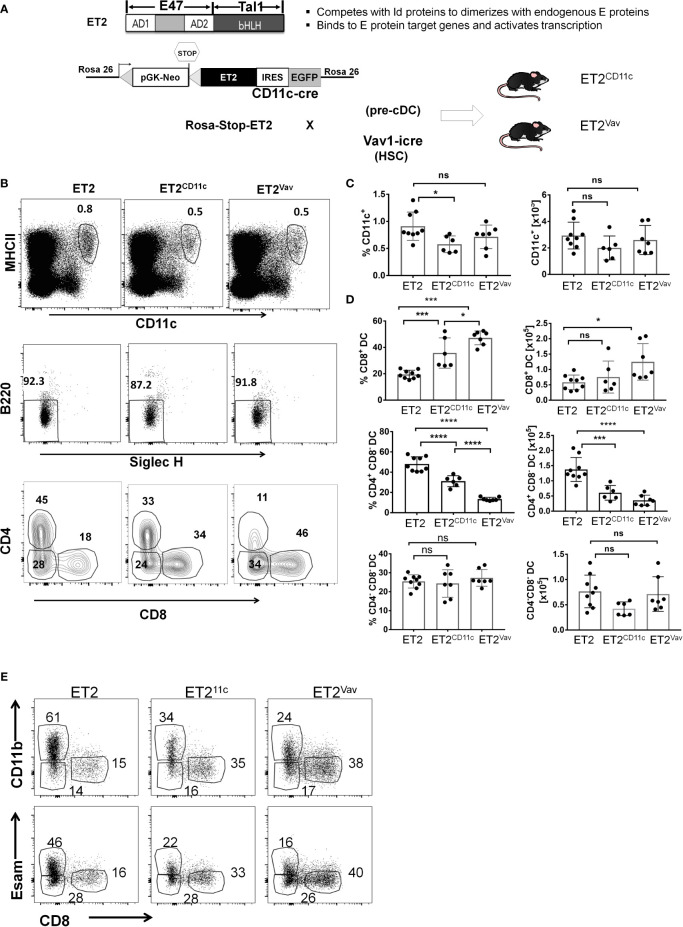
Impaired cDC2 development in the spleen. **(A)** Schematic diagrams of the ET2 chimeric protein and its properties (top) and the construction of the Rosa26 knock-in allele (ET2) crossed with indicated Cre transgenes (bottom). The designation of the resulting strains, ET2^CD11c^ and ET2^Vav^, are as labeled, and Cre is expressed in CD11c^+^ cells and total hematopoietic cells, respectively. **(B)** FACS analyses of splenocytes for indicated markers. Conventional dendritic cell (DC) subsets in the spleen were first defined as MHCII^hi^CD11c^hi^ cells, gated on SIGLECH^–^B220^–^ to exclude pDCs, and then fractionated based on CD4 and CD8 expression. Numbers indicate the percentages of the gated cells. **(C)** Average of the frequencies and total numbers of MHCII^hi^CD11c^hi^ cells. **(D)** Averages of the frequencies (within the MHCII^hi^CD11c^hi^ SIGLECH^–^B220^–^ fraction) and total numbers of the indic**a**ted subsets. Values from individual mice are shown, with the average indicated by the bar with SD. Data are pooled from three experiments. Significance was evaluated using a one-way ANOVA. *p < 0.05, **p < 0.01, ***p < 0.001, ****p < 0.0001. ns, not significant. **(E)** Analyses of splenic CD11c^+^B220^-^SIGLECH^-^ cells defined as in **(A)** for the expression of the markers as indicated. Strains of the mice are as described in **(A)**. Representative plots are shown.

To evaluate the impact of helix-loop-helix transcription factors in DC differentiation, we utilized our knock-in strain, ROSA26-Stop-ET2/EGFP (called ET2 hereafter), which expresses a chimeric protein, ET2, and EGFP *via* an IRES upon Cre-mediated deletion (**Figure 1A**) (18). ET2 contains the transcriptional activation domains of E47, a product of the E2A gene, and the DNA binding and dimerization domain of Tal1. ET2 does not form homodimers but has an affinity for endogenous E proteins that is similar to Id proteins, and thus can form heterodimers with endogenous E proteins and bind DNA (23, 24). Therefore, ET2, when ectopically expressed, can neutralize the effects of ID proteins such as ID2, but its activity is limited by the levels of endogenous E proteins (25). We crossed ET2 mice with either the CD11c-Cre knock-in allele (expressed in committed DCs and a fraction of pre-cDCs) or Vav1-iCre transgene (expressed in hematopoietic stem cells) to create the ET2^CD11c^ and ET2^Vav^ strains, respectively. EGFP expression in different relevant cell populations were determined as shown in **Supplemental Figure 1**. In steady state, these mice appear healthy without gross abnormalities.

Splenocytes of ~2 month old ET2^CD11c^ and ET2^Vav^ mice were analyzed along with controls that were ET2 mice without the Cre transgene. As shown in **Figure 1B**, DCs were first selected as CD11c^hi^MHCII^+^, a fraction which did not differ in numbers among the three strains (**Figure 1C**). Plasmacytoid DCs were excluded by a B220^–^SIGLECH^–^ gate. DCs were then separated by their expression of CD4 and CD8. The CD8^+^ population comprises cDC1s whereas the CD4^+^ subset represents cDC2s (**Figure 1B**). The CD4^–^CD8^–^ subset is also known to include cDC2s.

The frequency of the CD8^+^ cDC1 population was found to be significantly increased in both ET2^CD11c^ and ET2^Vav^ splenocytes compared to the controls, and the total number of cDC1s was also elevated in ET2^Vav^ splenocytes (**Figures 1B, D**). In contrast, the CD4^+^ cDC2 subset significantly decreased in percentage and number in both ET2^CD11c^ and ET2^Vav^ spleens. The proportion of the CD4^–^CD8^–^ cDC2 subset was not altered (**Figure 1D**). The expression of ET2 induced by the Vav1-iCre transgene had more profound effects on the numbers of cDC1s and cDC2s relative to cDC numbers in mice in which the ET2 expression was mediated by CD11c-Cre, which is turned on later in the hematopoietic hierarchy.

Furthermore, the characterization of cDC2s were confirmed with CD11b and ESAM markers. CD11c^hi^MHCII^+^B220^–^SIGLECH^–^ cells were gated on either of these markers together with CD8. The frequencies of CD11b^+^cDC2 and ESAM^+^ cDC2 were decreased similarly as CD4^+^cDC2 (**Figure 1E**). These results thus strengthened our conclusion as stated above.

Analyses of pDCs by gating on B220^+^SIGLECH^+^ cells, followed by gating for MHCII^+^CD11c^+^ cells, in the spleens of control, ET2^CD11c^ and ET2^Vav^ mice revealed only a modest reduction in the percentage but not in numbers of pDC in the spleen of ET2^Vav^ mice (**Supplemental Figure 2A**).

### Sustained E Protein Activity Leads to a Reduction in CD11b^+^CD24^hi^ cDC2s in the Lungs

**Figure 2:**
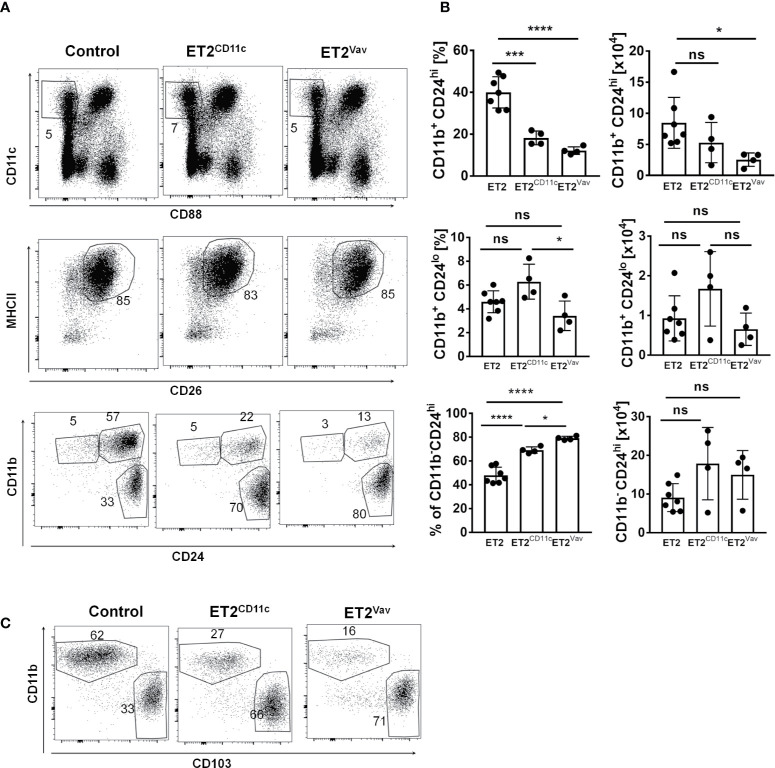
Impaired cDC2 development in the lung. **(A)** FACS analyses of lung resident dendritic cells from the indicated strains for the indicated surface markers. Numbers indicate the percentages of the gated cells. **(B)** The frequencies and total numbers of each dendritic cell (DC) subset (within the CD11c^+^MHCII^+^CD26^+^ fraction) are shown for individual mice, with the average and SD indicated by bars. Data are pooled from two experiments. Significance was evaluated using a one-way ANOVA. * p < 0.05, ***p < 0.001, ****P < 0.0001. ns, not significant. **(C)** Analyses of lung cells defined as in **(A)** except expression of the CD103 marker was determined in place of CD24. Strains of the mice are as described in **(A)**. Representative plots are shown.

Terminal differentiation of DCs in non-lymphoid tissue during homeostasis takes place in response to local tissue and environmental signals (26–28). Therefore, we evaluated lung resident cDC populations. As CD11b^+^ cells in the lung are heterogeneous, we used CD88 (which designates complement 5a receptor 1, C5aR1) to separate monocyte derived DC and macrophage populations from cDCs, as previously described by Nakano et al. (29). Next, we analyzed the CD11c^+^CD88^–^ subset for the expression of MHCII and CD26 (dipeptidyl peptidase-4 expressed by cDCs). MHCII^+^CD26^+^ cells were then dissected into cDC1 and cDC2 subsets using CD11b and CD24 or CD103 surface markers (**Figures 2A, C**).

The CD11b^+^ cells are considered cDC2s, within which CD11b^+^CD24^hi^ cells are more abundant and have previously been shown to be IRF4-dependent (30, 31). This subset decreased in percentage upon ET2 expression in both ET2^CD11c^ and ET2^Vav^ mice, but the numbers of these cells were significantly reduced only in ET2^Vav^ mice compared to the control (**Figure 2**). In contrast, no significant reduction in frequency or number was found in the IRF4-independent CD11b^+^CD24^lo^ subset. Likewise, the proportion of alveolar and resident macrophages in the lung was not significantly altered (**Supplemental Figure 3**). Taken together, these data show that ET2 expression specifically impairs the differentiation of the IRF4-dependent cDC2 subset in the lung.

### Early Expression of E Protein Leads to a Reduction in CD11b^+^ Pre-cDC2s

Since the impairment of cDC2 production was more severe in ET2^Vav^ compared to ET2 ^CD11c^ mice, we reasoned that the early induction of ET2 expression in bone marrow progenitors may impact cDC2 differentiation. Committed cDC precursors (CDP) are thought to give rise to two subsets of pre-cDC intermediates: pre-cDC1 and pre-cDC2 (8, 9, 11). These pre-cDCs circulate *via* blood to lymphoid and non-lymphoid tissues and undergo terminal differentiation in response to tissue and environmental signals.

**Figure 3:**
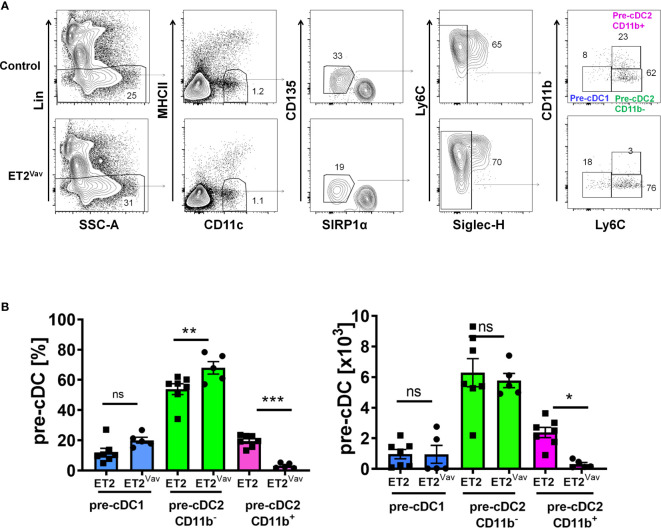
Defective pre-cDC2 specification in the bone marrow. **(A)** FACS analyses of bone marrow cells of ET2 and ET2^Vav^ mice. Total bone marrow cells were first depleted with lineage–specific antibodies against NK1.1, Ly6G, B220, CD3, CD19, and TER119. Lin^–^ cells were then analyzed sequentially for the expression of indicated markers. Final gates for the indicated subsets are as labeled. **(B)** Average percentages and numbers (mean ± SEM) of the indicated subsets (within the CD11c^+^CD135^lo^SIRPα1^-^SIGLECH^-^ fraction) in control and ET2^Vav^ mice were obtained by pooling data from two separate experiments. Significance was evaluated using one-way ANOVA. *p < 0.05, **p < 0.01, ***p < 0.001, ns, not significant.

To determine the frequencies of pre-cDCs in the bone marrow of control and ET2^Vav^ mice, we followed the scheme developed by Schlitzer et al. by first excluding granulocytes and B, T, NK and erythroid cells using a lineage cocktail containing antibodies against B220, CD19, CD3, NK1.1, Ly6G and TER119 (**Figure 3A**) (8). Within MHCII^–^CD11c^+^ cells, we gated the CD135^+^SIRPα^lo^ subset and further gated the SIGLECH-negative population. The resulting pre-cDC subset was then analyzed for the expression of CD11b and Ly6C to distinguish pre-cDC1s (CD11b^–^Ly6C^–^) and pre-cDC2s (Ly6C^+^) (**Figure 3A**). The pre-cDC2 population was previously defined as Ly6C^+^ (8), but we were able to further separate this group into CD11b^+^ and CD11b^–^ subsets. Within the pre-cDC pool, we detected no significant changes in pre-cDC1s (**Figure 3B**). Although the frequency of CD11b^–^pre-cDC2s was increased in ET2^Vav^ mice compared to control mice, the total numbers of this population were comparable (**Figure 3B**). In contrast, the frequency and number of CD11b^+^pre-cDC2s were both dramatically reduced by ET2 expression (**Figure 3B**).

### Expression of IRF4 and IRF8 in DC Precursors and Differentiated DCs

**Figure 4:**
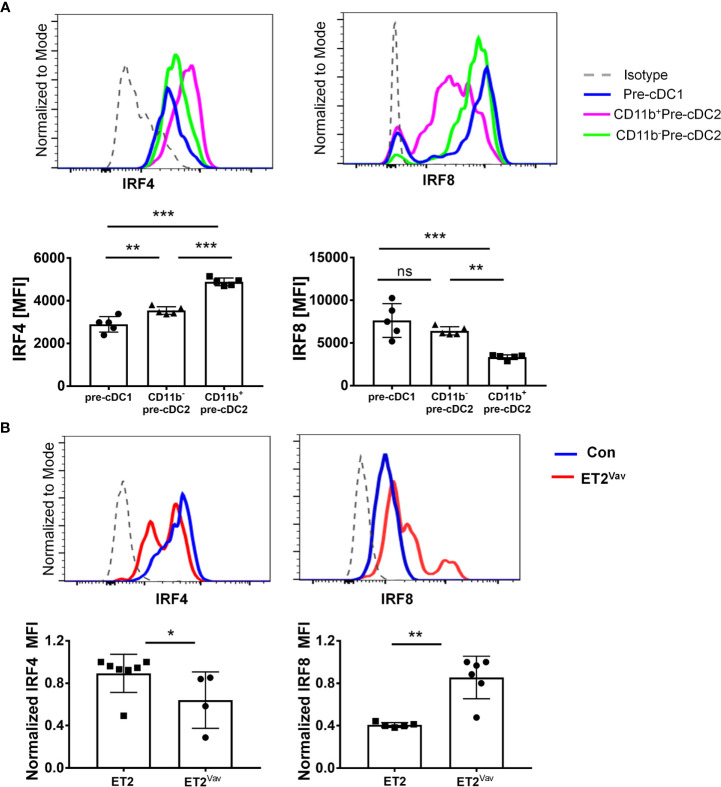
IRF4 and IRF8 expression *ex vivo.* **(A)** Intracellular staining of IRF4 or IRF8 was performed together with markers described in **Figure 3** for pre-cDC1 and pre-cDC2 subsets in the bone marrow of wild type mice as represented by the indicated colors. Histograms show the relative expression levels of IRF4 and IRF8. Isotype control antibodies were used as negative controls. **(B)** IRF4 and IRF8 expression in splenic CD4^+^cDC2s in control and ET2^Vav^ mice. Bar graphs show average MFIs. MFI values were normalized to the highest value in each experiment. Significance was evaluated using a Mann Whitney test. *p < 0.05, **p < 0.01, ***p < 0.001, ns, not significant.

IRF4 and IRF8 are required for development and function of cDC1s and cDC2s (11). They share common target genes and drive the expression of genes involved in DC function such as *H2-Ab1* (encoding MHCII), *Cd80*, *Cd86* and *Ccr7* but also have non-redundant roles in cDC specification. We first examined the expression of IRF4 and IRF8 proteins in the pre-cDCs in wild type bone marrow as defined in **Figure 3**. While pre-cDC1s and CD11b^–^pre-cDC2s contained low levels of IRF4 relative to the isotype control, CD11b^+^pre-cDC2 cells harbored notably higher levels of IRF4, consistent with the role of IRF4 in promoting cDC2 differentiation (**Figure 4A**). In contrast, pre-cDC1s and CD11b^–^pre-cDC2s expressed significantly higher levels of IRF8 compared to CD11b^+^pre-cDC2s (**Figure 4A**). These data suggest that attenuated IRF8 expression is necessary for differentiation of a subset of cDC2s.

When the levels of IRF4 and IRF8 were compared between ET2^Vav^ and control mice, we detected no differences in the expression of either transcription factor in pre-cDC1s and CD11b^–^pre-cDC2s (data not shown). Due to the extremely low frequency of CD11b^+^pre-cDC2s in ET2^Vav^ mice, we could not determine the levels of IRF4 and IRF8 with great confidence. However, we were able to measure the levels of IRF4 and IRF8 in splenic DCs in ET2^Vav^ mice. Although splenic cDC1s in ET2^Vav^ and control mice did not show differences in IRF4 or IRF8 levels (data not shown), CD11b^+^CD4^+^cDC2s exhibited changes in expression of these two proteins. Namely, the ET2-expressing cDC2s have lower levels of IRF4 but higher levels of IRF8 (**Figure 4B**), which may explain the cDC2 deficit in ET2 expressing mice.

### ET2 Impairs cDC2 Differentiation in Bone Marrow Cultures

**Figure 5:**
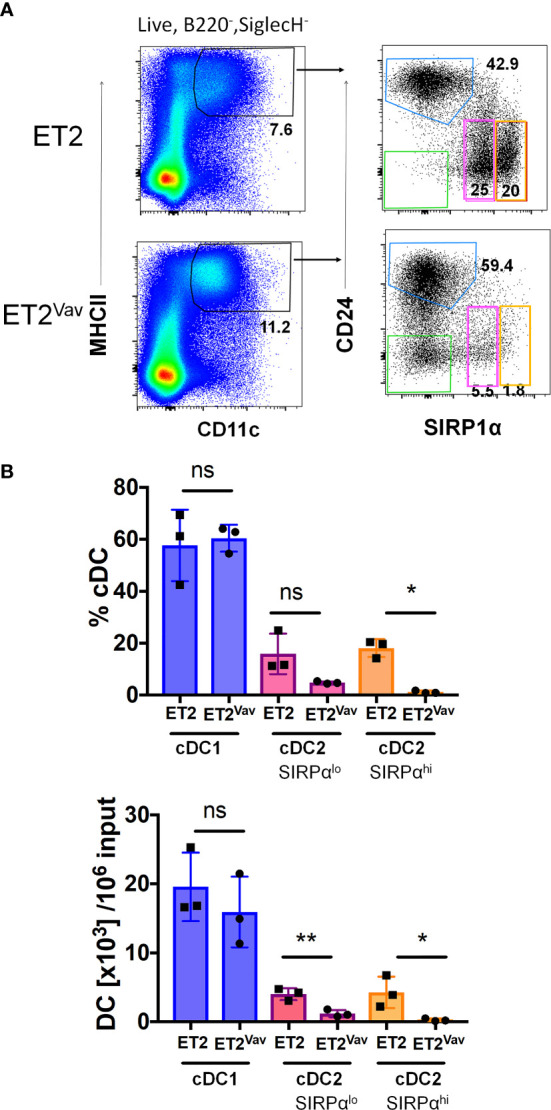
Inhibition of cDC2 differentiation *in vitro.* **(A)** FLT3 ligand supported dendritic cell (DC) cultures were initiated with lineage negative bone marrow cells from the indicated mouse strains. After nine days of culture, cells were harvested and analyzed by FACS. Live B220^–^SIGLECH^–^ cells were sequentially gated with the indicated markers. The cDC1 gate is indicated in blue, and cDC2 gates were outlined in pink (SIRPα^lo^) and orange (SIRPα^hi^). **(B)** Average percentages (within B220^-^ SIGLECH^–^CD11c^+^MHCII^+^ fraction) and numbers of the indicated subsets per 10^6^ input Lin- bone marrow cells or the indicated strains (mean ± SD). Statistical analyses were performed using a Student’s t test. *p < 0.05, ** p < 0.01, ns, not significant.

To complement our *ex vivo* analyses of DC development and closely monitor DC differentiation, we made use of a well-established *in vitro* culture system set up with bone marrow progenitors and supported by FLT3 signaling (32). Bone marrow cells from ET2^Vav^ and control mice were enriched for progenitors by performing lineage depletion with antibodies against CD11b, B220, CD5, Ly6G and TER119. Lineage-negative cells were then cultured in the presence of FLT3 ligand for 9 days before analyses using flow cytometry. Cultured cells were first selected as MHCII^+^CD11c^+^ and then scored for CD24 and CD172 (SIRPα) expression (19). cDC1s and cDC2s were defined as CD24^hi^SIRPα^–^ and CD24^lo^SIRPα^+^, respectively (**Figure 5A**). We noticed two subsets of CD24^lo^SIRPα^+^ cells that have lower and higher levels of SIRPα, and thus analyzed them separately. In addition, a population of CD24^–^SIRPα^–^ cells was readily detectable in ET2^Vav^ mice but almost absent in control mice; this population might represent intermediates that accumulated upon halted DC differentiation (**Figure 5**). As shown in **Figure 5B**, while the percentages and numbers of cDC1s produced were similar between the two strains of mice, the two subsets of cDC2s were significantly reduced in percentage and/or numbers by ET2 expression. These results suggest that ET2 impairs cDC2 differentiation from bone marrow progenitors.

In addition, pDC differentiation in the same cultures were also found to be impaired by ET2 expression (**Supplemental Figure 2B**). The frequency and numbers of pDCs were measured by gating on B220^+^SIGLECH^+^ cells, followed by gating for MHCII^+^CD11c^+^ cells. ET2 expression led to a dramatic reduction in pDC percentage and number in the cultures. However, the differentiation defect of pDCs and the underlying mechanism remain to be fully investigated, but this is beyond the scope of the current study.

### ET2 Diminishes cDC2 Transcriptional Programs

**Figure 6:**
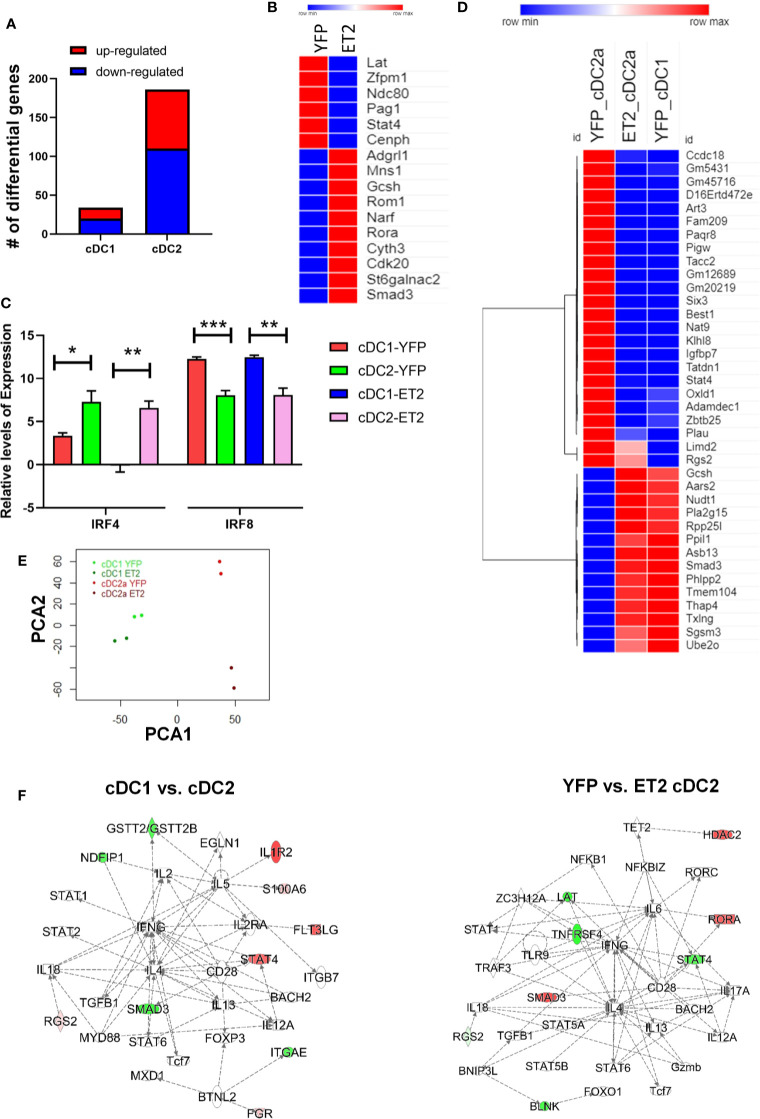
Analyses of the transcriptomes of splenic cDC1 and cDC2. Splenic cDC1s and CD4+cDC2s were isolated from two ET2^CD11c^ and YFP^CD11c^ mice and used for RNA sequencing. **(A)** Numbers of genes differentially expressed in indicated cells comparing YFP to ET2-expressing cells. **(B)** A select list of differentially expressed genes in cDC2 that match with putative E protein targets determined in T cells and group 2 innate lymphoid cells. **(C)** *Irf4* and *Irf8* expression in indicated subsets based on RNA sequencing data. Statistical analyses were performed using a one-way ANOVA, * < 0.05; ** p < 0.01 *** < 0.001. **(D)** A list of genes differentially expressed either comparing cDC1 and cDC2 or comparing YFP- to ET2-cDC2. **(E)** Ingenuity pathway analyses of the indicated differential gene sets. Principal component analysis of gene expression in indicated subsets. **(F)** Red indicates up-regulation and green shows down-regulation.

To further understand the mechanisms whereby augmented E protein activity impairs cDC2 differentiation, we determined the transcriptomes of CD8^+^cDC1 and CD4^+^cDC2 cells isolated from ET2^CD11c^ and YFP^CD11c^ mice. The YFP expressing mice served as proper controls for ET2^CD11c^ mice because the two strains express either EGFP or YFP driven by the CD11c-Cre. However, YFP^CD11c^ mice exhibited similar developmental profiles as ET2 mice lacking CD11c-Cre (data not shown). We used ET2^CD11c^ as opposed to ET2^vav^ mice to obtain sufficient cDC2s for RNA sequencing. Cells sorted from two individual mice of each strain were processed for RNA sequencing, and data were analyzed using standard bioinformatics tools. Comparing YFP to ET2-expressing cells, we detected 41 and 212 differentially expressed genes for cDC1 and cDC2, respectively (**Figure 6A** and **Supplemental Table 1**). These genes were selected using a cut off of a false discovery rate of less than 0.05. It is not surprising that ET2 expression led to fewer changes in gene expression in cDC1s because these cells possess much higher levels of ID2 than cDC2s, and ID2 could neutralize the effects of ET2.

We first assessed the effects of ET2 on potential E protein target genes. Since these genes in conventional dendritic cells are not known, we compared the differentially expressed genes with our gene sets found to be altered shortly after inducible ablation or addition of E proteins in T cell precursors (CD4 and CD8 double negative cells) or innate lymphoid cells (33). We found 8 out of the 34 protein coding genes in cDC1s and 29 out of 186 genes in cDC2s overlapped with a total of 2460 putative E protein-regulated genes detected in (33). Even with the distinct cell types included in the analysis, the intersections were deemed statistically significant using Fisher’s exact test (p <0.01 for cDC1 and p < 0.0046 for cDC2), suggesting that ET2 indeed alters E protein activity in cDCs. A select set of genes found in cDC2s are shown in **Figure 6B**.

We were intrigued that *Irf4* and *Irf8* were not among the lists of differentially expressed genes comparing YFP to ET2-expressing cells as in **Figure 6A**. When *Irf4* and *Irf8* expression was specifically examined, we still did not detect any difference between YFP to ET2-expressing cells but observed the expected distinct patterns comparing cDC1 and cDC2, namely high levels of IRF8 in cDC1 and IRF4 in cDC2 (**Figure 6C**). One possible explanation is that ET2-expression in ET2^CD11c^ splenic cDCs occurs after the pre-cDC stage, when E protein activity no longer influence *Irf4* and *Irf8* transcription. Although we detected alterations in IRF4 and IRF8 levels in splenic cDC2s of ET2^Vav^ mice (**Figure 4B**), these cells were present in much smaller numbers, possibly due to aberrant IRF4 and IRF8 levels in the pre-cDC stage.

To investigate additional transcriptional events that may contribute the impairment of cDC2 differentiation by ET2, we next focused on ET2-induced changes in gene expression in cDC2s. To determine the differences in the transcriptomes of cDC1 and cDC2, we compared gene expression in cDC1s and cDC2s from YFP^CD11c^ mice and obtained a total of 240 genes. The protein-coding genes from this list were then compared to the differentially expressed genes between YFP and ET2-cDC2s. Such analysis yielded 38 protein coding-genes that are altered by ET2 and also expressed differently in the two cDC subsets. The intersection is statistically highly significant (p < 2^-16^). Interestingly, the expression patterns of these genes in ET2-expressing cDC2s were similar to those of YFP-cDC1s but distinct from those of YFP-cDC2s, suggesting that ET2 diminished the transcriptome that is characteristic of cDC2s (**Figure 6D**). However, principle component analysis showed that ET2 cDC2 remained distinct from cDC1s even though they were markedly different from YFP cDC2s (**Figure 6E**).

Furthermore, ingenuity pathway analyses were performed on all of the genes differentially expressed comparing YFP-cDC1 to YFP-cDC2 and ET2-cDC2 YFP-cDC2, respectively. The top-ranking regulatory networks generated for both gene sets appeared similar, in that pathways centered on IL-4 and IFNG involved multiple transcription factors (**Figure 6F**). Among these, *Smad3* and *Stat4* exhibit opposite patterns of expression. Specifically, YFP-cDC2s have higher levels of *Stat4* and lower levels of *Smad3* mRNA compared to YFP-cDC1s. In contrast, the levels of *Stat4* and *Smad3* transcripts were lower and higher, respectively, in ET2-cDC2s relative to YFP-cDC2s. Therefore, STAT4 and SMAD3 may play critical roles in mediating the effects of ET2 in cDC2 differentiation.

## Discussion

We used a gain of function mutant of E protein transcription factors, ET2, to evaluate the role of E proteins in development of DCs in lymphoid and non-lymphoid tissue. The ET2 chimeric protein overcomes inhibition by ID proteins and forms heterodimers with endogenous E proteins and activates transcription of E protein targets. Using two Cre transgenes that begin to express at different stages in hematopoiesis, we assessed the timing of ET2 expression on DC differentiation in homeostasis. ET2^Vav^ mice, in which ET2 is expressed at the hematopoietic stem cell (HSC) stage, exhibited profound defects in cDC2 differentiation and an increase in cDC1s in the lung and spleen. Within the lung resident cDC2 pool, ET2 expression had the greatest effect on the CD11b^+^CD24^hi^ subset that is dependent on IRF4 for differentiation. ET2^CD11c^ mice showed a similar but less significant reduction in cDC2 numbers. This may stem from our observation that not all CD11c^+^ pre-cDCs showed evidence of Cre-mediated deletion in mice carrying only the Cre reporter (**Supplemental Figure 1**).

These results prompted us to investigate if ET2 impairs pre-cDC commitment in the bone marrow. Pre-cDC1 and pre-cDC2 subsets, thought to be derived from common DC progenitors (CDPs), differentiate to cDC1s and cDC2s in peripheral tissue (8). We fractionated the previously described pre-cDC2 population into CD11b^+^ and CD11b^–^ subsets. The CD11b^+^pre-cDC2 subset was dramatically diminished by ET2 expression whereas the CD11b^–^pre-cDC2 subset was slightly increased. Analyses of IRF4 and IRF8 expression in wild type mice revealed higher levels of IRF4 and lower levels of IRF8 in CD11b^+^pre-cDC2s, which suggests that these cells are destined to become cDC2s that are known to be dependent on IRF4. In contrast, CD11b^–^pre-cDC2s showed relatively higher levels of IRF8 and lower levels of IRF4, suggesting that they are less committed to the cDC2 fate and may represent earlier precursors. Alternatively, CD11b^–^pre-cDC2s may be a subset of pre-cDC2s whose differentiation is independent of IRF4 and resistant to ET2-mediated augmentation of IRF8 expression. Consistent with this, the ratio of IRF4 to IRF8 dictates the fate of human pre-cDC1s and pre-cDC2s (34).

In ET2^Vav^ mice, we detected little impact of ET2 on IRF4 and IRF8 expression in pre-cDC1s or CD11b^–^pre-cDC2s, and it was difficult to compare the levels of these transcription factors in CD11b^+^pre-cDC2s due to their scarcity in ET2-expressing mice. However, we did observe reduced levels of IRF4 and increased levels of IRF8 in splenic CD4^+^ cDC2s of ET2^Vav^ mice. This skewed ratio of IRF4 and IRF8 may be an underestimate of the effect of ET2 because if down-regulation of IRF8 and up-regulation of IRF4 is essential for cDC2 differentiation, the small numbers of cDC2s found in ET2^Vav^ mice may be selected as those that have relatively high levels of IRF4 and low levels of IRF8. The pre-cDC2s with high levels of IRF8 may not progress to the CD11b^+^ stage and differentiate to cDC2s in the spleen. Our data support the hypothesis that defects in cDC2 differentiation occur when pre-cDCs destined to become cDC2s either aberrantly upregulate or fail to downregulate IRF8.

It is well established that E protein transcription factors regulate *Irf8* expression (14, 35). An enhancer located 41 kb downstream of the transcriptional start site is known to contain several E boxes, to which E proteins bind. This enhancer was found to be utilized by another E protein, E2-2, in pDC, but a recent report shows that it is also instrumental for cDC1 specification from CDP (35). Deleting *Tcf3*, which encodes E2A, impairs the differentiation of both cDC1 and pDC (35). In contrast, gain of E protein function by ET2 expression leading to elevated IRF8 did not dramatically impact pre-cDC1 or cDC1 production, possibly because levels of IRF8 were already sufficient in these cells. However, the development of cDC2s is impaired by ET2 starting at the CD11b^+^pre-cDC2 stage, precursors that normally have low levels of IRF8 and high levels of IRF4. The scarcity of CD11b^+^pre-cDC2 cells in ET2^Vav^ mice prevented us from directly measuring IRF4 and IRF8 expression by using flow cytometry or RNA-sequencing in these cells. Nonetheless, we did detect modestly elevated IRF8 and reduced IRF4 protein levels in splenic cDC2s of these mice. The splenic cDC2s may, to some extent, include newly made cells from bone marrow progenitors, thus reflecting the features of CD11b^+^pre-cDC2s. Our data is consistent with the notion that increased E protein activity by ET2 results in elevated transcription of *Irf8* in CD11b^+^pre-cDC2s. Whether down-regulation of IRF8 is a pre-requisite for cDC2 specification and whether IRF8 interferes with certain specialized function of IRF4 are not entirely understood. In view of the report that ectopic expression of IRF8 blocks cDC2 differentiation (9), aberrant IRF8 expression may impede the IRF4-mediated specification of cDC2s through an unknown dominant-negative effect.

In addition, we were able to obtain sufficient splenic cDC1s and cDC2s from YFP^CD11c^ and ET2^CD11c^ mice for RNA sequencing and showed alterations of gene expression in cDC2s by ET2. These changes likely occurred in differentiated cDC1s and cDC2s since CD11-Cre induced ET2 expression only in a small subset of pre-cDC2s in the bone marrow (**Supplemental Figure 1A**). Interestingly, this gene set overlaps with the differential gene expression between cDC1 and cDC2, suggesting that the ET2-regulated genes are involved in determining the identity of cDC1 and cDC2. ET2 expression made the cDC2s adopt a transcriptome more characteristic of cDC1s and lose cDC2 specific genes. Ingenuity pathway analyses highlighted the top-ranking regulatory networks differentially expressed between cDC1s and cDC2s as well as between YFP-cDC2s and ET2-cDC2s. Both networks are centered on IL4 and IFNG and involve two transcription factors, STAT4 and SMAD3, which mediate cytokine signaling. Although STAT4 and SMAD3 may not be directly related, their levels of RNA are reciprocal. In cDC2s, STAT4 is increased and SMAD3 decreased relative to levels in cDC1s. However, ET2 expression in cDC2s down-regulated STAT4 and up-regulated SMAD3 levels, respectively. This is consistent with the down-regulation of *Stat4* and up-regulation of *Smad3* by E proteins in T cells (33). Importantly, *Stat4* transcription has been shown to be activated by ectopic expression of IRF4 but not IRF8 in *Irf4* ^-/-^ *Irf8* ^-/-^ bone marrow progenitors (36). STAT4 expression has also been shown to be dependent on Notch2 in cDC2s and on IRF4 on bone marrow derived DCs *in vitro* (37, 38). SMAD3 is not only downstream of TGF-beta signaling but also interacts with IL-37 and mediates its suppressive effects on pro-inflammatory cytokine production as well as dendritic cell activation (39, 40). Whether these transcription factors play crucial roles in cDC2 biology remains to be further investigated.

Taken together, findings from this study reveal the importance of tightly controlled E protein activities for DC development. The timing of E protein function and levels of E protein activities could bias numbers of distinct DC subsets and thus the immune responses they mediate. E protein activities can be regulated by the transcription of their genes, their ubiquitin-mediated degradation and by the levels of their naturally occurring inhibitors, ID proteins (12, 41, 42). Although ID2 is well-known for its essential role in cDC1 differentiation (16), the tonic levels of ID2 or other ID proteins at the CDP stage might also be important. A modest effect of E proteins on IRF8 expression may then be amplified by the ability of IRF8 to auto-regulate itself (11), which would lead to a more profound impact on DC differentiation.

## Data Availability Statement

The original contributions presented in the study are included in the article/**Supplementary Material**; further inquiries can be directed to the corresponding author.

## Ethics Statement

The animal study was reviewed and approved by the Institutional Animal Care and Use Committee at the Oklahoma Medical Research Foundation (OMRF).

## Author Contributions

SB, KT, CG, and YZ generated the data. SB, SK, CG, JDW, and X-HS analyzed the data and wrote the manuscript. All authors contributed to the article and approved the submitted version.

## Funding

This work was supported by grants from the NIH (1P20 GM103636-07) to X-HS and JW and (HL119501) to SK, and from the Presbyterian Health Foundation to X-HS. X-HS holds the Lew and Mira Ward Chair in Biomedical Research at the Oklahoma Medical Research Foundation.

## Conflict of Interests

The authors declare that the research was conducted in the absence of any commercial or financial relationships that could be construed as a potential conflict of interest.

## Supplementary Material

Supplementary Figure 1 EGFP expression in ET2^Vav^ and ET2^CD11c^ mice. **(A)** EGFP expression in splenic DCs and pre-cDCs (Lin^–^MHCII^+^CD11c^+^FLT3^+^SIRPα^l^<σπ >о</σπ > in the bone marrow of the indicated strains of mice is shown in histograms. CD4^+^, CD8^+^ and CD4^-^CD8^-^cDCs (SIGLECH^–^B220^–^MHCII^+^CD11c^hi^) are as defined in **Figure 1** whereas pDCs (SIGLECH^–^B220^+^MHCII^lo^CD11c^lo^) are as defined in **Supplemental Figure 2A**. **(B)** EGFP expression in bone marrow CDP, MDP and pre-pDC. The definition of the progenitors in lineage-negative bone marrow cells is shown on the top and EGFP levels in indicated progenitors are at the bottom.

Supplementary Figure 2 Impairment of pDC differentiation *in vitro* but not *in vivo.* **(A)** Splenic pDCs were analyzed by sequential gating for B220^+^SIGLECH^+^ and then for MHCII^+^CD11^+^ cells. Statistical analyses were performed using a one-way ANOVA, ** p<0.01. **(B)** Live cells generated *in vitro* as described in **Figure 5** were analyzed as described in **(A)**. Average percentages of live cells and total numbers of DCs per 10^6^ input Lin^–^ bone marrow cells are shown in bar graphs with SD. Statistical analyses were performed using a Student’s t test. **** p<0.0001.

Supplementary Figure 3 ET2 expression does not impact macrophage differentiation. FACS analyses of lung resident macrophages (rM) and alveolar macrophages (AM) were performed on the indicated strains with the indicated markers. Numbers indicate the percentages of the gated cells. Definition of AM and rM are as indicated. Values from individual mice are shown, with the average indicated by the bar with SD. Data are pooled from three experiments. Significance was evaluated using a one-way ANOVA. * p<0.05, ns, not significant.

Supplementary Table 1 Lists of differentially expressed genes.
