# Supplementary figures and images for: Augmenting E Protein Activity Impairs cDC2 Differentiation at the Pre-cDC Stage
Source: Front Immunol. 2020 Dec 18;11:577718. doi: 10.3389/fimmu.2020.577718 (PMC7775562; doi:10.3389/fimmu.2020.577718)

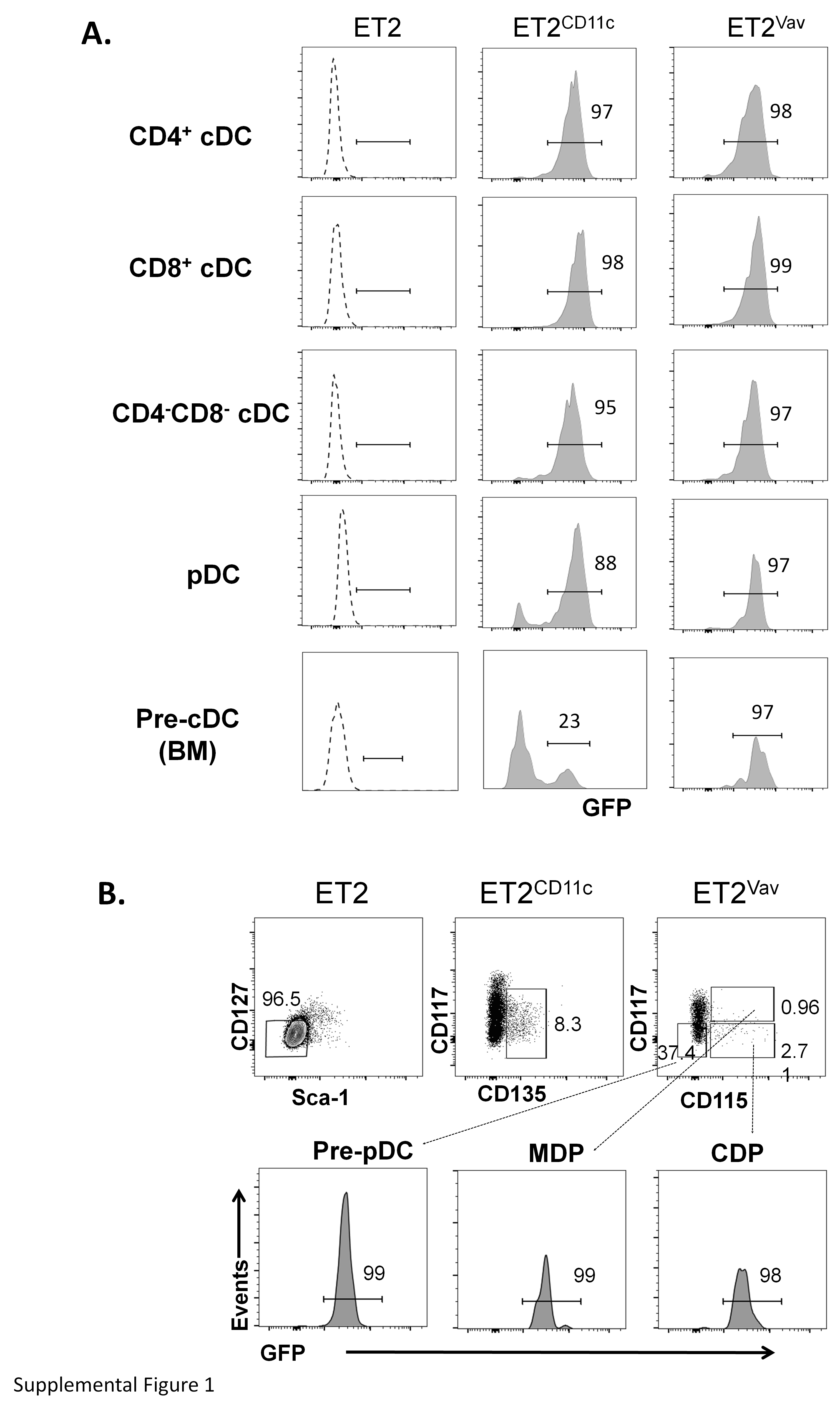

Supplement: Supplementary Figure 1 — EGFP expression in ET2Vav and ET2CD11c mice. (A) EGFP expression in splenic DCs and pre-cDCs (Lin–MHCII+CD11c+FLT3+SIRPαl<σπ >о</σπ > in the bone marrow of the indicated strains of mice is shown in histograms. CD4+, CD8+ and CD4-CD8-cDCs (SIGLECH–B220–MHCII+CD11chi) are as defined in Figure 1 whereas pDCs (SIGLECH–B220+MHCIIloCD11clo) are as defined in Supplemental Figure 2A . (B) EGFP expression in bone marrow CDP, MDP and pre-pDC. The definition of the progenitors in lineage-negative bone marrow cells is shown on the top and EGFP levels in indicated progenitors are at the bottom. [file Image_1.tif]

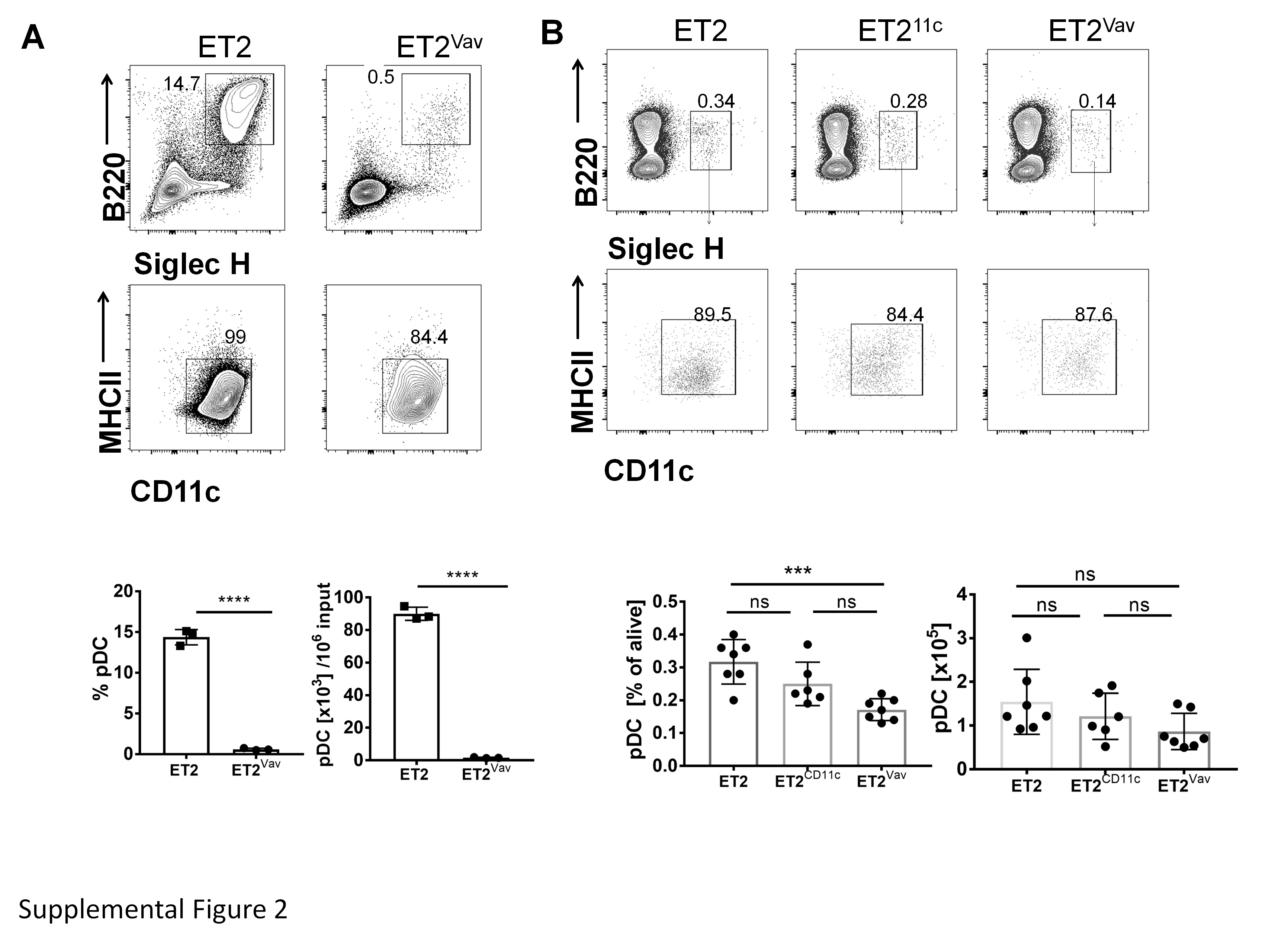

Supplement: Supplementary Figure 2 — Impairment of pDC differentiation in vitro but not in vivo. (A) Splenic pDCs were analyzed by sequential gating for B220+SIGLECH+ and then for MHCII+CD11+ cells. Statistical analyses were performed using a one-way ANOVA, ** p<0.01. (B) Live cells generated in vitro as described in Figure 5 were analyzed as described in (A). Average percentages of live cells and total numbers of DCs per 106 input Lin– bone marrow cells are shown in bar graphs with SD. Statistical analyses were performed using a Student’s t test. **** p<0.0001. [file Image_2.tif]

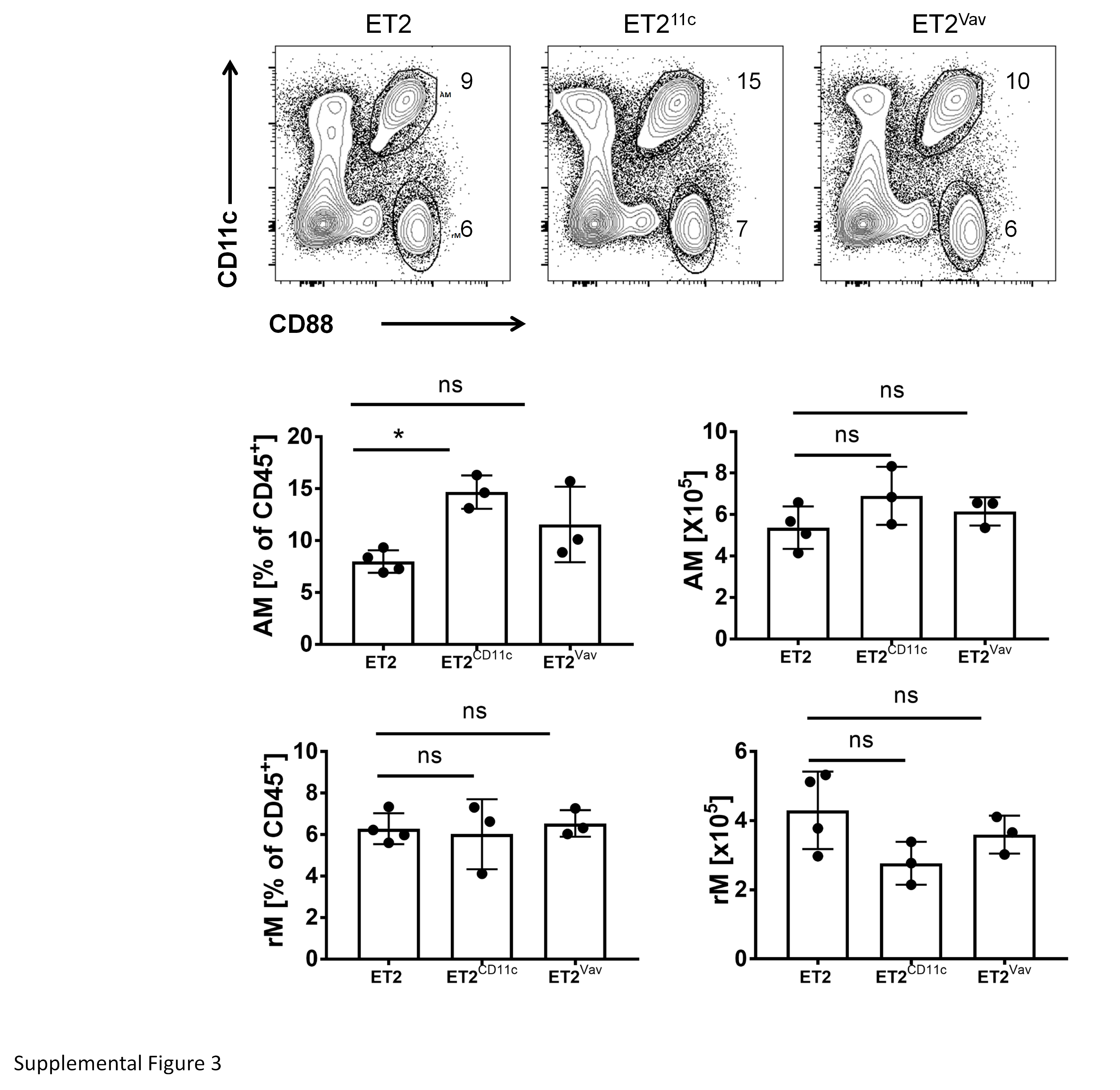

Supplement: Supplementary Figure 3 — ET2 expression does not impact macrophage differentiation. FACS analyses of lung resident macrophages (rM) and alveolar macrophages (AM) were performed on the indicated strains with the indicated markers. Numbers indicate the percentages of the gated cells. Definition of AM and rM are as indicated. Values from individual mice are shown, with the average indicated by the bar with SD. Data are pooled from three experiments. Significance was evaluated using a one-way ANOVA. * p<0.05, ns, not significant. [file Image_3.tif]
